# Surgical Treatment of Spinal Metastases–A Retrospective Single-Center Study of 268 Patients

**DOI:** 10.3390/jcm14238308

**Published:** 2025-11-22

**Authors:** Bernhard Springer, Christoph Stihsen, Josef G. Grohs, Anna Rienmüller, Philipp Funovics, Petra Krepler, Reinhard Windhager

**Affiliations:** Department of Orthopedics and Trauma Surgery, Medical University of Vienna, 1090 Vienna, Austria; bernhard.springer@meduniwien.ac.at (B.S.); christoph.stihsen@meduniwien.ac.at (C.S.); josef.grohs@meduniwien.ac.at (J.G.G.); anna.rienmueller@meduniwien.ac.at (A.R.); philipp.funovics@meduniwien.ac.at (P.F.); petra.krepler@meduniwien.ac.at (P.K.)

**Keywords:** spinal metastases, spinal surgery, survival after surgical treatment of spinal metastases, surgical outcome

## Abstract

**Background/Objectives**: Cancer is the second leading cause of death, and spinal metastases may occur in up to 40% of patients with cancer. The purpose of the current study is to evaluate survival after surgical treatment of spinal metastases, and to identify risk factors that might shorten postoperative survival. **Methods**: Two-hundred sixty-eight patients who underwent surgery due to spinal metastases at a single center between 1990 and 2019 were evaluated retrospectively. Various variables and prognostic scores were analyzed to assess their impact on postoperative survival. **Results**: Two-hundred thirty-three patients (86.9%) died with a mean time from surgery to death of 14.84 months. Twelve months postoperatively, the survival rate of the entire cohort was 41.8%. Patients who were 65 years or older and were bedbound or in a wheelchair had a significantly shorter survival rate (*p* = 0.007). Further risk factors for a worse survival rate were: higher preoperative ASA-score (β = 2.19, 95% CI 1.34–3.57; *p* = 0.002), higher age at the time of surgery (β = 1.03, 95% CI 1.00–1.05; *p* = 0.041), presence of preoperative additional extraspinal bone metastases (β = 1.15, 95% CI 1.01–3.76; *p* = 0.047), ambulatory status (β = 1.21, 95% CI 1.04–1.4; *p* = 0.013), and elevated CRP-value > 5 mg/dL (β = 3.02, 95% CI 1.59–5.73; *p* = 0.001). **Conclusions**: Patients who are 65 years or older and bedbound or in a wheelchair had a significantly shorter survival period. When considering treatment options for patients with spinal metastases, a higher ASA-score, a higher age at the time of surgery, the presence of preoperative additional extraspinal bone metastases, being bedbound or in a wheelchair, and an elevated CRP-value > 5 mg/dL should be considered as significant risk factors for a worse survival rate. Available prognostic scores may only predict a patient’s survival accurately in the short-term follow-up.

## 1. Introduction

In the United States, cancer is the second leading cause of death [1]. Up to 66% of patients who suffer from cancer develop metastases, and approximately 70% of patients with a malignant tumor show metastatic disease at the time of death [2,3]. Spinal metastases may occur in up to 40% of patients with cancer [4]. Due to more effective treatment options and longer survival, the incidence of spinal metastases is increasing continuously [5,6,7]. It has been shown that spinal metastases occur most frequently in the thoracic spine (70%), followed by the lumbar spine (20%), and ultimately the cervical spine (10%) [8,9,10]. Intramedullary metastases may occur on rare occasions [11,12,13,14]. In 10 to 40% of cases, multiple lesions occur at non-contiguous levels [8,9,10,15]. Any malignant tumor may develop spinal metastases [1]. Metastases may present as osteolytic or osteoblastic lesions [16]. The most frequent underlying primary tumors are breast, lung, prostate, and renal cell cancer, respectively (60% of spinal metastases) [17,18,19,20]. In approximately 5% of patients, the underlying primary malignancies are gastrointestinal tumors. A review of Wong et al. [21] postulated spinal metastases in 30% of patients with renal cell cancer, in 45% of patients with lung cancer, in 75% of patients with breast carcinoma, and in 90% of patients with prostate cancer.

Patients with spinal metastases commonly suffer from back pain [6]. Approximately 10 to 20% of patients with spinal metastases show neurological deficits due to spinal cord compression [9]. Constans et al. [16] analyzed 600 patients with neurological deficits due to spinal metastases and showed that the onset of neurological symptoms may vary from acute (within less than 48 h) to insidious (slow progress over an interval of up to one month). The management of spinal metastases is discussed controversially in the orthopedic and neurosurgical field [1]. Treatment options include chemotherapy, hormonal therapy, radiation, and surgery [4]. Improvements in therapy options led to an increased survival of patients [22]. Indications for a surgical approach are unbearable pain, progression of neurological deficits, an unknown histological diagnosis, and instability of the spine [22]. Furthermore, patient life expectancy should exceed three months [4,23]. Prediction of the prognosis is crucial when evaluating the best treatment option for patients with spinal metastases [24]. As a result, various prognostic scoring systems have been designed to evaluate life expectancy and probability of death preoperatively. Tomita et al. [25], Tokuhashi et al. [26,27], and Bauer and Wedin [28] each postulated a prognostic scoring system. Schoenfeld et al. [29] also designed the “New England Spinal Metastases Score” (NESMS). Dependent on the score, different parameters are considered. As a result, each scoring system might calculate a different survival prognosis and, therefore, suggest a different treatment strategy for the same patient [24]. The Global Spine Tumour Study Group (GSTSG) provided a prediction tool that predicts the probability of death within three, six, 12, and 24 months, respectively [30].

Inaccurate prediction of patient survival affects the decision process and, consequently, the postoperative outcome [31]. The purpose of the current study is to evaluate survival after surgical treatment of spinal metastases and to identify further risk factors that might shorten the postoperative survival.

## 2. Materials and Methods

After obtaining institutional review board approval (study # 2330/2016), all consecutive patients who underwent surgery due to spinal metastases and unbearable pain, impending instability of the spine, or neurological deficits between January 1990 and December 2019 at the Department of Orthopedics of the Medical University of Vienna were retrospectively assessed. Three hundred twenty-five patients were identified. Overall, 57 patients were excluded. Nine patients were excluded because the primary malignant tumor was located at the spine. Twelve patients received a kyphoplasty and were excluded. Twenty- six patients had incomplete medical records, and in ten cases the metastases were located sacral. After exclusion, 268 patients were available for evaluation. All patients were preoperatively evaluated by an anesthesiologist and classified according to the physical status classification of the American Society of Anesthesiologists (ASA) [32]. All patients who presented with neurological deficits underwent neurological assessment by a neurologist following institutional standard operating procedures. Demographic data, clinical information, laboratory blood tests, and comorbidities were collected from the medical reports of each patient. The primary tumor and existing extraspinal metastases (location and number of metastases) were also collected. Regarding spinal metastases, we collected the affected vertebral level. The histological report of each patient was also collected to verify that all included patients received surgery due to spinal metastases. Furthermore, the date of death, the reason for death, and the time from surgery to death were collected for all included patients. Information regarding additional therapies like pre- or postoperative radiation or chemotherapy were also collected. The following functional and prognostic scores were retrospectively assessed and calculated for all patients: Frankel mobility score [33], Karnofsky performance status scale [34], Bauer score [28], GSTSG-Score [30], Tomita score [25], NESMS [29], and Tokuhashi [26,27].

Surgical and conservative treatment options for patients with spinal metastases have been improved substantially over the last few decades [35]. To take these therapeutical adaptions into account, we evaluated survival outcomes for each decade (1990s, 2000s and 2010s) separately.

All patients received dorsal or ventral fusions or decompression surgeries without fusion. Depending on bone quality, extent of osteolysis, and existing spinal instability, fusions were performed up to three levels above and below the affected vertebral body. In cases with an insufficient screw fixation, the fixation was augmented with cement.

### Statistical Analysis

Frequencies, proportions, means, ranges, and standard deviations were used to describe the study cohort. The Kolmogorov–Smirnov test was used to analyze variables for normality. As not all variables met the criteria for normal distribution (*p* < 0.001 to 0.2) the Mann–Whitney U test was used to compare the distribution of independent variables between two groups. More than two groups were compared using the Kruskal– Wallis test. To compare nominal variables the chi-square test was used. Finally, the Kaplan–Meier analysis was used to evaluate patient survival. The log rank test was used to compare the survival between various independent factors. Cox regression was used to evaluate if independent risk factors for a shorter survival were detectable. All calculations were per- formed with SPSS software for Mac OS X (Version 23.0; IBM, Armonk, NY, USA). Results with a *p*-value < 0.05 were considered statistically significant.

## 3. Results

The study cohort consisted of 111 female patients (41%) and 157 male patients (59%) with a mean age at the time of surgery of 60.4 years (range 17–86 years). Mean body mass index (BMI) was 24.53 kg/m2 (range 16.46–37.04). Ninety-eight patients (36.5%) were admitted to the hospital due to neurological symptoms (motoric deficits (n = 40, 14.9%), sensorial deficits (n = 36, 13.4%), incomplete paraplegia (n = 14, 5.2%), complete paraplegia (n = 8, 3.0%)). One hundred sixty-one patients (59.9%) were admitted due to unbearable pain and nine patients (3.3%) due to other reasons. In 199 patients (74%), dorsal stabilization surgery with decompression was performed. In 30 patients (11.2%), decompression surgery or laminectomy was performed, and 39 (14.5%) patients underwent ventral stabilization of the spine. All patients were preoperatively evaluated by an anesthesiologist and classified according to the ASA physical status classification [32]. Six patients (2%) were classified as ASA 1, 103 (38%) as ASA 2, 157 (59%) as ASA 3, and 2 (1%) as ASA 4. Overall, the mean follow-up was 18.2 months (range 0 – 207.8 months). One-hundred fifty-five patients (57.8%) died within the first year. These patients had a mean follow-up of 4.1 months. Patients that died after the first 12 months after surgery or patients who were still alive (113 patients, 42.2%) had a mean follow-up of 23.99 months.

In 41 patients (15.3%), metastases were located in the cervical spine. One hundred twenty-five patients (46.6%) had thoracic metastases, and in 97 patients (36.2%) metastases were located in the lumbar spine. Table 1 shows the distribution of the primary tumors. Table 2 shows the distribution of the extraspinal metastases of all the included patients, in detail.

Overall, 79 (29.5%) complications occurred within a mean time to complication of 1.7 months (range: 0–75.7 months). Eighteen patients (22.8%) had surgical related complications. Two patients (2.5%) had an infection, 16 patients (20.3%) had postoperative bleeding, 14 patients (17.7%) showed a postoperative neurological deficit, eight patients (10.1%) had a leakage of the dura mater, six patients (7.6%) had a seroma, four patients (5.1%) had a postoperative hematoma, two patients (2.5%) suffered postoperative pneumonia, seven patients (8.9%) had other internal complications and two patients (2.5%) had other complications. In 46 of these patients (58.2%), revision surgery was performed. As shown in Table 3, the occurrence of complications was not a risk factor for worse postoperative survival.

### 3.1. Survival Analysis

**Figure 1:**
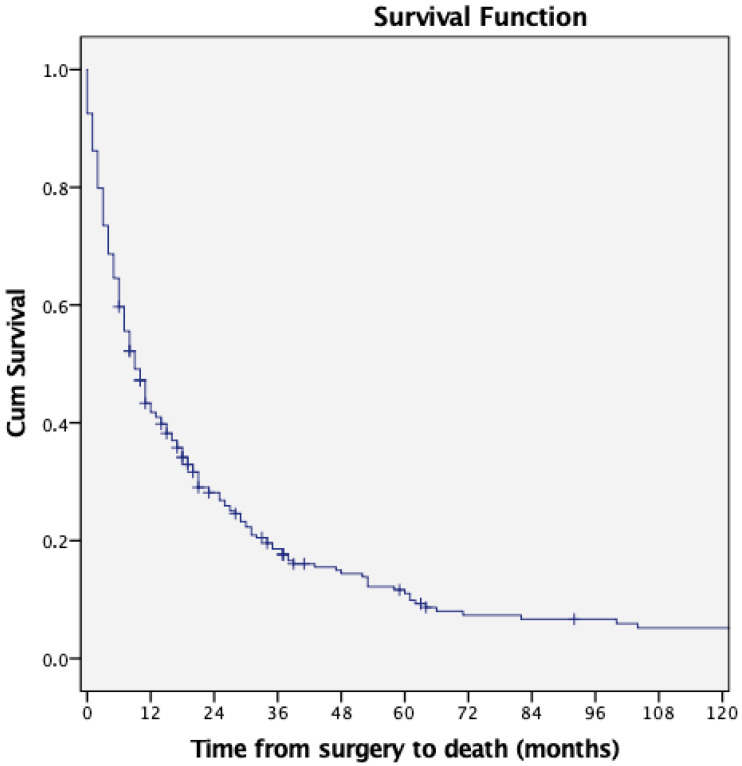
Overview of the overall survival rate of the entire cohort.

Two hundred thirty-three patients (86.9%) died within a mean time of 14.84 months (range: four days to 207 months) after surgery. At the time of death, patients had an average age of 61.5 years (range: 21 years to 86 years). The Kaplan–Meier survival analysis revealed that 12 months after surgery, 41.8% of the patients were still alive. The survival rates after 24, 48 and 60 months were 28.1%, 14.4% and 11.1%, respectively. Figure 1 shows the overall survival of all patients postoperatively. There was a significant difference in survival between the various primary tumor entities (*p* < 0.001). Cox multivariate regression revealed that the ASA score (β = 2.19, 95% CI, 1.34–3.57; *p* = 0.002), age at the time of surgery (β = 1.03, 95% CI, 1.00–1.05; *p* = 0.041), the presence of preoperative additional extraspinal bone metastases (β = 1.15, 95% CI, 1.01–3.76; *p* = 0.047), ambulatory status (β = 1.21, 95% CI, 1.04–1.4; *p* = 0.013), and an elevated CRP value > 5 mg/dL (β = 3.02, 95% CI, 1.59–5.73; *p* = 0.001) are significant risk factors for shorter survival. Table 3 shows the results of the Cox multivariate regression analysis in detail.

The Kaplan–Meier log-rank test showed that patients with additional extraspinal bone metastases had a significantly shorter survival rate (means: 17.08 vs. 9.93 months, *p* = 0.006). Survival was also significantly shorter for patients with a higher preoperative ASA classification (ASA I: 43.6 months, 95% CI 10.04–77.17; ASA II: 17.75 months, 95% CI 13.89–21.60; ASA III: 11.81 months, 95% CI 8.07–15.55; ASA IV: 0 months; *p* < 0.001). Patients with a CRP value > 5 mg/dL also showed shorter survival compared to patients with a lower CRP value (7.68 vs. 13.78, *p* = 0.005).

**Figure 2:**
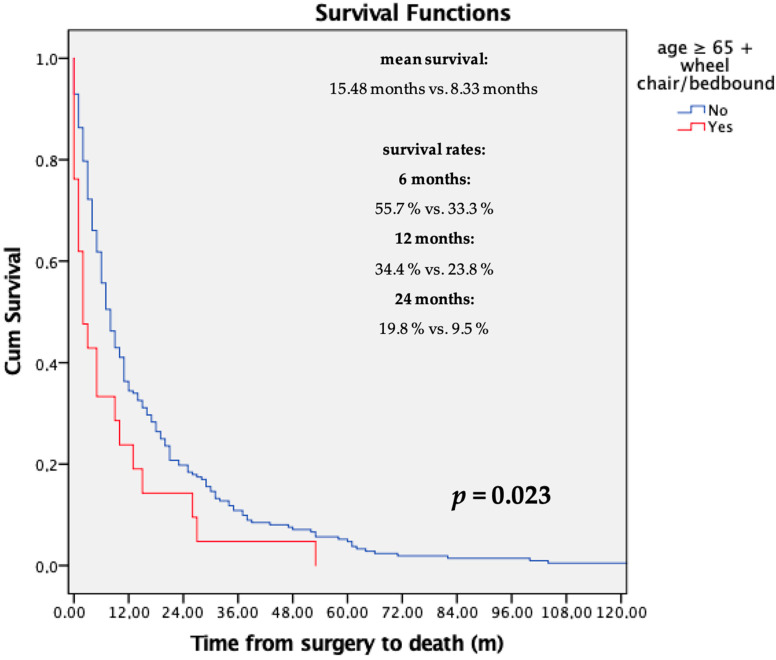
Overview of the survival rate of patients who were ≥ 65 years and bedbound or in a wheelchair.

Our data showed that patient age affects survival significantly, since patients who were 65 years or older had a significantly shorter survival rate compared to younger patients (mean survival: <65 years: 16.94 months; ≥65 years: 11.62 months; *p* = 0.028). Detailed analyses regarding ambulatory status were performed. A significantly shorter survival rate was observed in patients who were in a wheelchair or bedbound, compared to patients who were still able to walk with or without a walking aid (means: 10.20 vs. 16.07 months; *p* = 0.037). As a result, patients who fulfilled both of the above-mentioned criteria also had significantly shorter postoperative survival (means: 8.33 vs. 15.48 months; *p* = 0.023). Figure 2 shows the exact survival rate after 6, 12, and 24 months, respectively.

We also compared the survival rate between the decades in which surgery was performed. Patients who received surgery in the 2010s survived for a significantly shorter time than patients who received surgery in the 1990s or 2000s. The ASA score in the 2000s was significantly lower than in the 2010s. However, the mean ASA score in the 2010s was comparable to the mean ASA score in the 1990s. There was no difference regarding complication rates between the three decades. However, significantly more revision surgeries were performed in the 2010s compared to the 1990s and 2000s. Table 4 shows the results in detail.

### 3.2. Analysis of Prognostic Scores

Several scores were published to estimate the survival chances of patients over a certain period. We compared the actual survival of the included patients with various prognostic scores. We divided the patients into four groups, dependent on time of death (within three months after surgery, four to six months after surgery, seven to 12 months after surgery and 13 to 24 months after surgery). Seventy-one patients (26.5%) died within the first three months after surgery. These patients had significantly worse prognostic scores and a significantly higher probability of death in the GSTSG score compared to patients who were still alive. Patients who died between the fourth and sixth postoperative month showed a significantly higher result in the Karnofsky index compared to patients who were still alive at that time. The Tomita score was significantly higher in patients who died (5.03 vs. 4.41, *p* = 0.048). All other evaluated scores did not differ between patients who survived and patients who died. Seven to 12 months postoperatively the Bauer score and the Tomita score showed significantly lower results in patients who died. Interestingly, the NESMS was significantly higher in patients who did not die (2.54 vs. 1.91, *p* = 0.003). The probability of death in the GSTSG score was significantly higher in patients who actually died (53.53 vs. 39.43, *p* < 0.001). There were no differences in any prognostic score between patients who died after the 12th postoperative month and patients who were still alive at that time. Table 5, Table 6, Table 7 and Table 8 show the results in detail.

## 4. Discussion

The most important finding of the current study is that patients who were 65 years or older and were in a wheelchair or bedbound had a significantly worse postoperative survival rate. Our data also showed that the preoperative ASA score, age at the time of surgery, the presence of preoperative additional extraspinal bone metastases, ambulatory status, and an elevated CRP value > 5 mg/dL were significant risk factors for a worse survival rate. According to our findings, available prognostic scores may predict a patient’s survival accurately within the first postoperative year. However, they hardly allow an accurate long-term prediction for survival after the first postoperative year.

Overall survival in our cohort 12 months postoperatively was 41.8%. Previously published papers have shown similar survival rates 12 months postoperatively ranging from 38% to 59% [36,37,38,39]. Various risk factors have been published, which might shorten the postoperative survival after surgical intervention due to spinal metastases. De Meue et al. [31] evaluated the influence of various laboratory values. They found that albumin was associated with a longer survival rate and CRP was associated with a shorter survival rate. In our current study, albumin did not have an influence on survival. However, we also identified an increased CRP value > 5 mg/dL as risk factor for a shorter postoperative survival rate. Various studies showed that the entity of the primary tumor affected the survival rate [25,26,36,40,41,42,43]. Even though we observed a significantly different survival rate between the various tumor entities, the entity of the primary tumor was not detected as risk factor for a shorter survival rate in our cohort. Another aspect that affects postoperative survival is patient age at the time of surgery [44,45,46]. Patients older than 65 years were also associated with a higher complication rate [44]. On the other hand, it has been shown that even patients who are older than 80 years might benefit from surgery in case of spinal metastases [47]. In our opinion, it is very difficult to decide which patient surgery should be performed and who should no longer be considered as a candidate for surgery. However, our data showed that patients who were older than 65 years had a significantly shorter postoperative survival rate. When combined with their preoperative ambulatory status, the difference in the mean survival rate was 8.3 months versus 15.48 months. There were relatively few patients who fulfilled both criteria (being 65 years or older and being bedbound or in a wheelchair). Nevertheless, due to the great difference in the survival rate, we suggest considering these two combined factors when discussing the best treatment option for a patient with spinal metastases.

Patients with cancer and spinal metastases are vulnerable to postoperative complications. Therefore, careful patient selection and treatment selection is necessary [48]. Reliable prognostic tools that predict a patient’s survival rate appropriately are crucial to finding the best treatment option. In 1990, Tokuhashi et al. [27] were the first to propose such a prognostic scoring system. Since then, these prognostic tools have had a strong influence on the treatment of patients with spinal metastases [49]. However, the current literature shows that only a few of the available scores are consistently reliable or practically useful in predicting patient survival [50,51,52,53,54]. While Cassidy et al. [51] suggested preferably using the Bauer score, Schoenfeld et al. [49] validated the NESMS and stated that it is able to predict patient survival to a significantly higher degree than the Tokuhashi or Tomita score. Kim et al. [55] evaluated the Tomita and Tokuhashi score. They stated that the Tomita score predicted survival more accurately than the Tokuhashi score. They also suggested performing surgery in patients with a Tomita score ≤ 8 and a Tokuhashi score ≥ 6. According to our data, the Tomita score was the only score that showed significantly different scores between patients who died and patients who were still alive at every evaluated time up to 12 months postoperatively. The Bauer score, the NESMS, the Tokuhashi score and the GSTSG score did not show a significant difference at some of the evaluated moments. Especially in the prolonged follow-up over 12 months, there was not a single score that showed a significant difference in the score result between patients who died and patients who survived. Therefore, we conclude that the available scores might predict short-term survival accurately, but they hardly allow an accurate prediction of survival after the first postoperative year.

In general, it is accepted that surgical intervention might be considered in patients who have a predicted survival rate longer than three months [23]. The decision to perform surgery on patients with spinal metastases is difficult and should be made based on various factors. It is important to identify patients who benefit substantially from surgical intervention [38]. Fehlings et al. [39] performed a prospective multicenter study with 142 patients who underwent surgery due to a single symptomatic metastatic epidural spinal cord compression lesion. They postulated that surgery provides sustained and immediate relief of pain, improvement in quality of life, and improvement in neurological and functional outcomes in patients who have a survival prognosis of at least three months, while only an acceptable risk exists. Orenday-Barraza et al. [56] evaluated the ten-year trend in the surgical management of patients with spinal metastases and postulated an advantage of surgical intervention with adjuvant radiation to improve tumor control, neurological function, and overall survival. They also stated a trend towards less invasive spine procedures. However, the basic idea of stabilization and decompression remains the cornerstone in the surgical treatment of spinal metastases. Another study by Wagner et al. [57] coincided with the previously mentioned findings and postulated that in case of spinal metastases, the first step should be decompression and dorsal stabilization. In general, due to the wide heterogeneity, an interdisciplinary treatment strategy is crucial [57]. Beside surgical interventions, radiosurgery has been supported as an efficient and safe treatment option [58]. However, it has also been shown that approximately 33% of conservative cases needed surgical intervention within the first year [59]. Our findings coincide with the generally accepted consensus that further prospective studies are still needed to identify reliable risk factors that are easy to evaluate in the clinical setting, and that provide consistent results.

### Limitations

The current study has the following limitations: (1) Our study is a retrospective study with a heterogeneous cohort and the common disadvantages that are associated with the retrospective study design. However, there are only a few papers with a comparable number of included patients that focus on the outcome after surgery due to spinal metastases. To minimize bias, we excluded patients whose medical records were incomplete. (2) The study includes patients from 1990 to 2019. During this period, revolutionary changes in the treatment of spinal metastases were achieved regarding surgical and conservative treatment options. To take these therapeutical adaptions into account, we evaluated the survival rate of each decade separately. (3) One might argue that the two characteristics “patients who are 65 years or older” and “being bedbound or in a wheelchair” were chosen arbitrarily. The current study does not aim to provide specific cut-off values. However, our data suggest that patients who fulfill both characteristics had a significantly worse postoperative survival rate. (4) Another limitation is that the various primary tumor entities were not evaluated separately. However, the number of patients per tumor entity was relatively small. Due to that fact, we decided to evaluate the entire cohort together to get more expressive results.

## 5. Conclusions

Patients who are 65 years or older and bedbound or in a wheelchair had a significantly shorter survival rate. When considering treatment options for patients with spinal metastases, a higher ASA score, a higher age at the time of surgery, the presence of preoperative additional extraspinal bone metastases, being bedbound or in a wheelchair, and an elevated CRP value > 5 mg/dL should be considered as significant risk factors for a worse survival rate. Available prognostic scores may predict patient survival accurately within the first postoperative year. However, they hardly allow an accurate long-term prediction of survival after the first postoperative year.

## Figures and Tables

**Table 1:** Overview of the primary tumor entities.

Primary Tumor	Number of Patients (%)	Survival in Months (95% CI)	75 Percentile [Months]
Renal	72 (26.9%)	16.19 (12.20–20.18)	26.00
Thyroid	11 (4.1%)	20.90 (6.05–35.75)	35.50
Mamma	37 (13.8%)	25.56 (15.60–35.53)	34.00
Lung	39 (14.6%)	10.11 (5.22–15.00)	9.25
Uterus/Cervix	9 (3.4%)	10.78 (3.30–18.25)	19.50
Colon	11 (4.1%)	11.50 (4.80–18.20)	20.75
Liver	7 (2.6%)	8.42 (0–18.09)	18.00
Gastric	2 (0.7%)	1.00 (0–2.96)	N/A
Myeloma	5 (1.9%)	23.00 (23.00–23.00)	23.00
Prostate	12 (4.5%)	13.58 (8.55–18.62)	20.75
Other known tumors	56 (20.9)	12.61 (3.25–22.00)	11.00
Unknown primary tumor	7 (2.6%)	2.40 (0–5.35)	5.50

Data is shown as absolute and relative values. Survival is presented as mean values with 95% confidence interval (CI). Other known tumors include: melanoma, anal carcinoma, pancreas carcinoma, esophagus carcinoma, lymphoma.

**Table 2:** Overview of the additional extraspinal metastases.

Location	Number of Patients (%)
Extraspinal bone metastases	85 (31.9%)
Lung metastases	68 (25.4%)
Liver metastases	33 (12.3%)
Other abdominal metastases	43 (16.0%)
Brain metastases	7 (2.7%)

Data is shown as absolute and relative values.

**Table 3:** Overview of the Cox regression analysis.

Variable	β-Value (95% CI)	*p*-Value
Sex	1.323	0.280
	(0.796–2.198)	
Body mass index (kg/m^2^)	0.942	0.055
	(0.887–1.001)	
Primary tumor	1.052	0.277
	(0.960–1.152)	
Age at surgery	1.025	0.041 *
	(1.001–1.049)	
Reason for admission	0.972	0.782
	(7.95–1.188)	
Preoperative extraspinal bone metastases	1.949	0.047 *
	(1.010–3.760)	
Mobility	1.207	0.013 *
	(1.040–1.401)	
Pre-/postoperative chemotherapy	1.312	0.362
	(0.732–2.351)	
Radiation of the spine preoperatively	0.717	0.413
	(0.324–1.589)	
Preoperative radiation	1.248	0.591
	(0.556–2.802)	
Postoperative radiation	0.695	0.154
	(0.422–1.904)	
Albumin level	1.182	0.539
	(0.693–2.015)	
CRP-value > 5 mg/dL	3.015	0.001 *
	(1.586–5.731)	
Decreased hemoglobin level preoperatively	0.877	0.649
	(0.499–1.542)	
Perioperative complications	1.123	0.665
	(0.663–1.713)	

Survival is presented as odds ratio with 95% confidence interval (CI). *: statistically significant results. CRP: C-reactive protein. mg/dL: milligrams per deciliter.

**Table 4:** Comparison between decades.

Variable	1990s	2000s	2010s	*p*-Value (90s vs. 00s)	*p*-Value (90s vs. 10s)	*p*-Value (00s vs. 10s)
Survival	18	16.6	8.1	0.809	<0.001 *	0.002 *
(months)	(12.7–23.3)	(11.8–21.3)	(5.3–10.8)			
ASA	2.64	2.38	2.69	0.002 *	0.348	<0.001 *
	−0.48	−0.56	−0.57			
Complication rate	33.30%	30.40%	24.70%	0.677	0.334	0.612
Revision rate	10.40%	16.50%	24.70%	0.239	0.01 *	0.1

Survival is shown as mean values with 95% confidence interval (95% CI). The ASA classification is shown as mean values with standard deviation (SD). Complication and revision rates are presented as relative values. *: statistically significant results.

**Table 5:** Overview when comparing the prognostic scores between patients who died and patients who were still alive 3 months postoperatively.

	Dead ≤ 3 Months (n = 268)		
	Yes (n = 71; 26%)	No (n = 197; 74%)	
Variable	Mean (SD)	Mean (SD)	*p*-Value
Karnofsky Index	0.00 (16.90)	75.43 (16.86)	0.006 *
Bauer Score	2.23 (0.91)	2.77 (0.91)	<0.001 *
New England Spinal Metastasis Score	1.46 (1.23)	2.31 (0.95)	<0.001 *
Tokuhashi Score	8.49 (2.52)	10.36 (2.6)	<0.001 *
Tomita Score	5.66 (2.24)	4.53 (1.9)	<0.001 *
GSTSG Score	20.1 (10.39)	16.34 (10.7)	0.001 *

*: statistically significant results

**Table 6:** Overview when comparing the prognostic scores between patients who died and patients who were still alive 4–6 months postoperatively.

	Dead 4–6 Months (n = 197)		
	Yes (n = 37; 19%)	No (n = 160; 81%)	
Variable	Mean (SD)	Mean (SD)	*p*-Value
Karnofsky Index	80.27(15.18)	74.31 (17.07)	0.014 *
Bauer Score	2.62 (1.01)	2.81 (0.89)	0.272
New England Spinal Metastasis Score	1.94 (1.12)	2.38 (0.91)	0.110
Tokuhashi Score	10.16 (2.59)	10.40 (2.61)	0.513
Tomita Score	5.03 (1.92)	4.41 (1.93)	0.048 *
GSTSG Score	30.83 (15.78)	28.53 (15.26)	0.350

*: statistically significant results

**Table 7:** Overview when comparing the prognostic scores between patients who died and patients who were still alive 7–12 months postoperatively.

	Dead 7–12 Months (n = 160)		
	Yes (n = 47; 29%)	No (n = 113; 71%)	
Variable	Mean (SD)	Mean (SD)	*p*-Value
Karnofsky Index	71.70 (17.59)	75.40 (16.80)	0.175
Bauer Score	2.41 (0.91)	2.96 (0.83)	<0.001 *
New England Spinal Metastasis Score	1.91 (0.97)	2.54 (0.83)	0.003 *
Tokuhashi Score	9.89 (2.69)	10.58 (2.56)	0.159
Tomita Score	5.02 (2.21)	4.18 (1.77)	0.016 *
GSTSG Score	51.53 (17.82)	39.43 (17.17)	<0.001 *

*: statistically significant results

**Table 8:** Overview when comparing the prognostic scores between patients who died and patients who were still alive 13–24 months postoperatively.

	Dead 13–24 Months (n = 113)		
	yes (n = 34; 30%)	no (n = 79; 70%)	
Variable	Mean (SD)	Mean (SD)	*p*-Value
Karnofsky Index	77.94 (16.29)	74.30 (17.00)	0.243
Bauer Score	2.88 (0.95)	3.00 (0.78)	0.707
New England Spinal Metastasis Score	2.37(0.89)	2.59 (0.81)	0.199
Tokuhashi Score	10.79 (2.41)	10.48 (2.63)	0.614
Tomita Score	4.56 (2.3)	4.01 (1.46)	0.526
GSTSG Score	56.71 (17.60)	53.10 (18.43)	0.168

## Data Availability

The data presented in this study are available on request due to privacy and legal aspects.
